# CD44 Promotes Lung Cancer Cell Metastasis through ERK–ZEB1 Signaling

**DOI:** 10.3390/cancers13164057

**Published:** 2021-08-12

**Authors:** Yen-Yun Wang, Anupama Vadhan, Ping-Ho Chen, Yen-Lung Lee, Chih-Yeh Chao, Kuang-Hung Cheng, Yu-Chiuan Chang, Stephen Chu-Sung Hu, Shyng-Shiou F. Yuan

**Affiliations:** 1 Translational Research Center, Kaohsiung Medical University Hospital, Kaohsiung 807, Taiwan; wyy@kmu.edu.tw; 2 Department of Medical Research, Kaohsiung Medical University Hospital, Kaohsiung 807, Taiwan; 3 School of Dentistry, College of Dental Medicine, Kaohsiung Medical University, Kaohsiung 807, Taiwan; phchen@kmu.edu.tw; 4 Center for Cancer Research, Kaohsiung Medical University, Kaohsiung 807, Taiwan; 5 Graduate Institute of Medicine, College of Medicine, Kaohsiung Medical University, Kaohsiung 807, Taiwan; u105800008@kmu.edu.tw; 6 Department of Surgery, Kaohsiung Medical University Hospital, Kaohsiung 807, Taiwan; l63228322@gmail.com; 7 Department of Mechanical Engineering, National Pingtung University of Science and Technology, Pingtung 912, Taiwan; cychao@mail.npust.edu.tw; 8 Institute of Biomedical Sciences, National Sun Yat-Sen University, Kaohsiung 804, Taiwan; khcheng@faculty.nsysu.edu.tw (K.-H.C.); ycc@mail.nsysu.edu (Y.-C.C.); 9 Department of Medical Laboratory Science and Biotechnology, Kaohsiung Medical University, Kaohsiung 807, Taiwan; 10 Department of Dermatology, College of Medicine, Kaohsiung Medical University, Kaohsiung 807, Taiwan; stephen@kmu.edu.tw; 11 Department of Dermatology, Kaohsiung Medical University Hospital, Kaohsiung 807, Taiwan; 12 Department of Obstetrics and Gynecology, Kaohsiung Medical University Hospital, Kaohsiung 807, Taiwan

**Keywords:** CD44, lung cancer, ERK, ZEB1, metastasis

## Abstract

**Simple Summary:**

Lung cancer treatment has always been challenging as its metastasis rate is higher than other cancers. Cancer stemness promotes cancer metastasis, and it is important to understand the mechanism behind the cancer-stemness-mediated metastasis. CD44 is a well-known cancer stem cell marker and plays a role in tumor metastasis in different cancer types. In this study, we investigated the role of CD44 in lung cancer metastasis by using clinical studies as well as in vitro and in vivo studies. We found that CD44 promotes the migration and invasion abilities of lung cancer cells through ERK–ZEB1 signaling. This study provided evidence that CD44 may be a possible therapeutic target to decrease lung cancer metastasis.

**Abstract:**

Lung cancer is a malignancy with high mortality worldwide, and metastasis occurs at a high frequency even when cancer spread is not detectable at primary operation. Cancer stemness plays an important role in malignant cancer behavior, treatment resistance, and cancer metastasis. Therefore, understanding the molecular pathogenesis behind cancer-stemness-mediated metastasis and developing effective approaches to prevent metastasis are key issues for improving cancer treatment. In this study, we investigated the role of CD44 stemness marker in lung cancer using in vitro and clinical studies. Immunohistochemical staining of lung cancer tissue specimens revealed that primary tumors with higher CD44 expression showed increased metastasis to regional lymph nodes. Flow cytometry analysis suggested that CD44 positive cells were enriched in the metastatic lymph nodes compared to the primary tumors. CD44 overexpression significantly increased migration and invasion abilities of lung cancer cells through CD44-induced ERK phosphorylation, ZEB1 upregulation, and Claudin-1 downregulation. Furthermore, ERK inhibition suppressed the migration and invasion abilities of CD44-overexpressing lung cancer cells. In summary, our in vitro and clinical results indicate that CD44 may be a potential prognostic and therapeutic marker for lung cancer patients.

## 1. Introduction

Lung cancer is the most commonly diagnosed cancer as well as the leading cause of cancer death in males and the second leading cause of cancer death in females worldwide [1,2]. Among newly diagnosed lung cancer patients, 26% of patients showed mediastinal lymph node metastasis, and 49% of patients showed extrathoracic metastasis. The most common metastasis sites in non-small cell lung cancer (NSCLC) patients are the adrenal gland, liver, brain, and bone [3]. NSCLC patients may develop recurrent or metastatic disease even after complete surgical resection, and there are multiple factors that influence patient survival following disease recurrence. In a series of 1073 NSCLC patients, recurrence was identified in 445 patients (41%) after complete resection, with a median time of 11.5 months to recurrence following surgery and a median survival of 8.1 months following recurrence [4]. In addition, most lung cancer patients are diagnosed at an advanced stage. Therefore, it is very important to understand the underlying mechanisms of lung cancer metastasis.

CD44, a non-kinase transmembrane cell surface glycoprotein, belongs to the class of cell adhesion molecules (CAMs) [5], which are expressed in embryonic stem cells, connective tissue, and bone marrow. CD44 is a well-known cancer stem cell marker and is upregulated in certain cancer cell subpopulations [6]. CD44 variant isoforms (CD44v) are generated by alternative splicing, and these isoforms contain 10 constant exons and different combinations of the remaining variant exons [7]. For example, human CD44 is encoded by 19 exons, among which 10 exons are constant in all isoforms. Hyaluronic acid (HA), a major component of the extracellular matrix (ECM) expressed by both stromal and cancer cells, is the main ligand for CD44, and an HA-binding domain is present in all CD44 isoforms [8]. The binding of HA to the CD44 ligand-binding domain induces a conformational change in the intracellular domain of CD44, which promotes the interaction of the intracellular domain with adaptor proteins and cytoskeletal elements. This leads to the activation of different signaling pathways, resulting in cell proliferation, migration, and invasion [9].

Cancer metastasis is a complex process in which multiple key events take place, including epithelial cancer cell-to-mesenchymal cancer cell transition (EMT), angiogenesis induction, cancer cells intravasation and extravasation, regaining of epithelial traits (mesenchymal-to-epithelial transition, MET), and finally, formation of a metastatic tumor in the distant microenvironment [10]. Mounting evidence indicates that many malignancies are maintained by cancer stem cells (CSCs), which are a subpopulation of cells that display stem cell properties and contribute to treatment resistance and metastasis [10]. The role of CD44 in promoting tumor metastatic ability has been studied in different cancer types. In breast cancer and cervical cancer, high CD44-expressing cells promote EMT through twist [11], and in pancreatic cancer, CD44 promotes cancer cell invasion ability through membrane-bound metalloproteinase (MT1-MMP) [12]. In colon cancer, CD44 overexpression is associated with mesenchymal phenotype and increased cell migration and invasion abilities, while CD44 knockdown decreases cell migration and invasion abilities [13]. Additionally, the interaction of CD44 with chitinase-like protein-like 1 (CHI3L1) promotes gastric cancer metastasis through the β-catenin/Erk/Akt signaling [14]. In prostate cancer, CD44 promotes migration and invasion of cancer cells through Hippo-Yap signaling.

Overexpression of CD44 and its isoforms has been reported to be associated with poor prognosis in gliomas [15], breast cancer [16], pancreatic cancer [17], lung cancer [18], prostate cancer, and head and neck carcinoma [19]. CD44 has also been reported to promote cancer stemness, aggressiveness [20,21,22], and drug resistance in different cancer types [23,24,25,26]. Herein, we report that high CD44 expression promotes lung cancer cell metastasis in vitro and in vivo through activation of ERK–ZEB1 signaling.

## 2. Materials and Methods

### 2.1. Patient Samples

The lung cancer tissue samples were obtained from Kaohsiung Medical University Hospital from patients undergoing surgery (IRB no: KMUH-IRB-20130272). Overall survival (OS) is defined as the time period between the date of diagnosis and death. Informed consent was obtained from all patients.

### 2.2. Tissue Sections and Immunohistochemistry

Tissue section slides were prepared from formalin-fixed and paraffin-embedded tissue blocks. Two pathologists reviewed hematoxylin-eosin stained sections independently to select representative areas of tumor or normal region.

### 2.3. Cell Culture

Human lung cancer cell lines A549, H1299, CL1-0, and H441 were purchased from the Bioresource Collection and Research Center (BCRC, Hsinchu, Taiwan). A549 cells were maintained in DMEM F12 medium, H1299 in DMEM, and CL1-0 and H441 in RPM1 1640 medium with 5% CO_2_ at 37 °C in a humidified incubator. All culture media were supplemented with 10% FBS (fetal bovine serum) and 1% PSA (penicillin G, streptomycin, and amphotericin B).

### 2.4. XTT Colorimetric Assay

Cell proliferation rate was determined by the XTT (2,3-Bis(2-methoxy-4-nitro-5-sulfophenyl)-2H-tetrazolium-5-carboxanilide) colorimetric cell proliferation assay (Roche Molecular Biochemicals, Potsdam, Germany). Briefly, 3000 cells were seeded in a 96-well plate in full growth medium. At 24, 48, and 72 h after seeding, medium was replaced with XTT solution containing PMS (N-methyl dibenzopyrazine methyl sulfate) (1:1000 dilution) and incubated for 30 min. Optical density (OD) was measured at 490 nm and 650 nm [27].

### 2.5. Transwell Migration and Invasion Assays

Cell migration assay was carried out using Transwell (Corning Costar Corp., Cambridge, MA, USA) membrane filter inserts in 24-well tissue culture plates with 6.5 mm diameter and 8μm pore size. Following CD44 overexpression or knockdown, lung cancer cells were trypsinized and suspended in serum-free medium. The cells were then seeded on the upper chamber of Transwell, and FBS-containing medium was added to the lower chamber. After 24 h of incubation at 37 °C, crystal violet was used to stain the cells. Non-migrated cells from the upper chamber were removed, and migrated cells were imaged by Oympus100 and analyzed by using Image J software. For invasion assay, BioCoat Matrigel (Corning, Bedford, MA, USA) transwell inserts were used, and the procedure was similar to transwell migration assay and according to the manufacturer’s instructions.

### 2.6. Flow Cytometry for Cell Cycle Analysis and CD44 Expression

Trypsinized cells were fixed in 1 mL of 70% ethanol overnight at 4 °C, centrifuged at 1500 g for 5 min, and resuspended in 1 mL PBS (phosphate-buffered saline) solution containing 50 µg/mL RNase and 50 µg/mL propidium iodide (Sigma Chemical Co. 3050 Spruce Street, St. Louis, MO, USA). Stained cells were analyzed by using flow cytometry (BD, Becton, Dickinson & Co, San Diego, CA, USA).

For study of CD44 expression in cancer tissues, fresh primary lung tumors and metastatic lymph nodes were dissociated by ophthalmic scissors, followed by incubation with digestion buffer (150 U/mL collagenase and 50 U/mL hyaluronidase in serum-free DMEM medium) at 37 °C for 16 h. FBS was added to a final concentration of 10% to stop the reaction, followed by centrifugation at 500× *g* for 5 min. After removal of supernatant, 2 mL RBC lysis buffer (0.15 M NH4Cl, 10 mM KHCO3, 0.1 mM Na2EDTA, pH 7.2) was added to digest RBC. After centrifugation at 500× *g* for 5 min and removal of the supernatant, we added 2 mL medium and 2 mL Ficoll-liquor followed by centrifugation at 700× *g* for 5 min to separate and aspirate the cells at the grayish-white layer, and washed them with DMEM medium followed by centrifugation at 500× *g* for 5 min. A total of 2 × 10^7^ cells were added with anti-CD45 magnetic beads (11153D, Thermo Fisher Scientific, Invitrogen, Waltham, MA, USA) and incubated at 4 °C for 30 min. At last, the cells were put onto magnetic platform for 10 min and harvested for flow cytometry analysis (1 × 10^5^ cells). Before flow cytometry analysis, the cells were labeled with fluorochrome-conjugated antibodies against CD44 (11-0441-82, Thermo Fisher Scientific, eBioscience, Waltham, MA, USA) and CD24 (45-0242-82, Thermo Fisher Scientific, eBioscience manufacturer, Waltham, MA, USA) [28].

### 2.7. Western Blot

The effects of CD44 overexpression and knockdown on expression of EMT markers, including epithelial marker Claudin-1 and mesenchymal marker ZEB1, were determined by using Western blot. Antibodies for CD44, α-tubulin, p-ERK 1/2, ERK, ZEB1, and Claudin-1 were purchased from GeneTex (US). Mitogen-activated protein kinase inhibitor PD98059 was purchased from Sigma (St. Louis, MO, USA).

### 2.8. In Vivo Metastasis Mouse Model

For distant (lung) metastasis in NOD SCID mouse model, lung cancer A549 cells with CD44 knockdown were resuspended in PBS (1 × 10^5^ cells/100 μL in PBS) and injected into the tail vein of mice. After 8 weeks, the mice were sacrificed, and mice lungs were collected and fixed in 10% formalin. To check for lung metastasis, hematoxylin and eosin (H&E) were used to stain lung tissue sections, and lung nodules were quantified by a dissecting microscope. Animal experiments were approved by the animal ethics committee of our hospital (IACUC number: 109029).

### 2.9. Statistical Analysis

Student’s *t*-test and two-sided χ^2^ test methods were used to determine statistical significance. All *p*-values less than 0.05 were considered statistically significant.

## 3. Results

### 3.1. High Expression of CD44 in Primary Tumors and Lymph Nodes Is Associated with Poor Survival of Lung Cancer Patients

**Figure 1:**
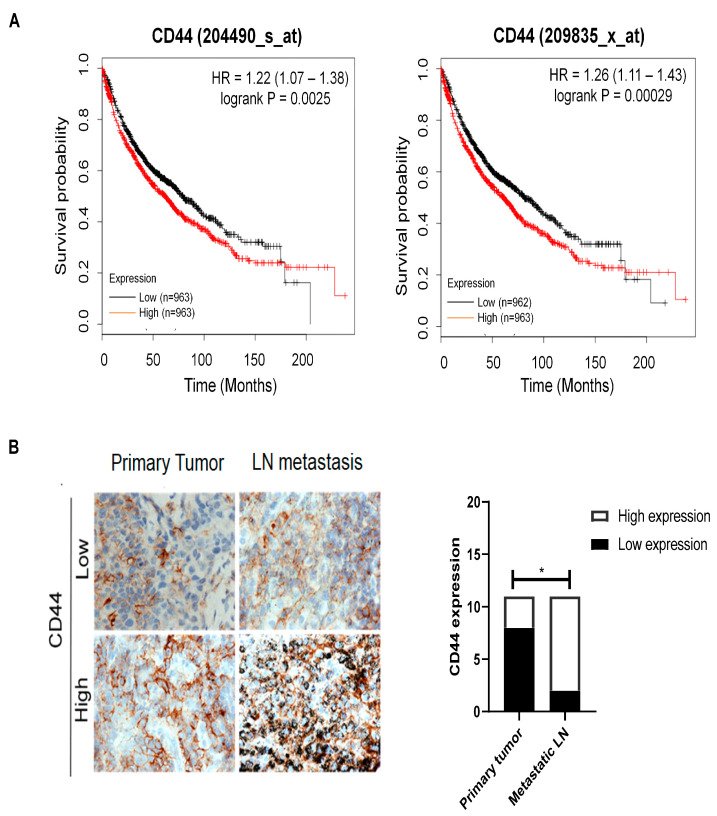

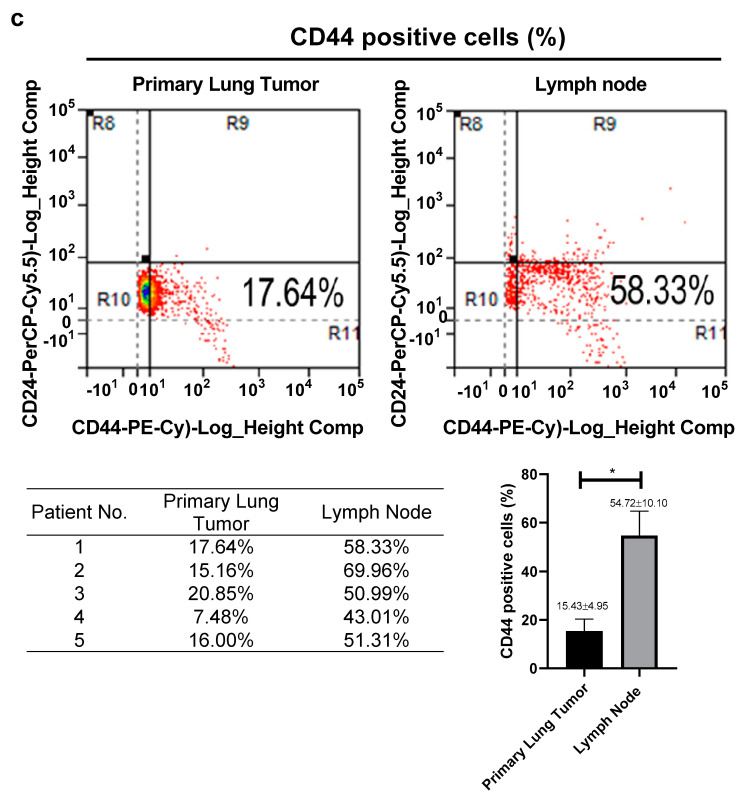
CD44 is overexpressed in primary tumors and lymph nodes, and is correlated with poor survival. (**A**) Kaplan–Meier analysis of OS, according to the expression of CD44 mRNA by using lung cancer online microarray datasets software. (**B**) Representative images of IHC for CD44 expression in primary tumors and lymph node metastases (**left**) and bar graph showing the expression of CD44 in primary tumors and metastatic lymph nodes of lung cancer patients (**right**). (**C**) Flow cytometry results showing the percentage of CD44 positive cells in primary tumors and metastatic lymph nodes analyzed by flow cytometer (**left**) and bar figure showing the average of CD44 positive cells in primary lung tumors and metastatic lymph nodes (**right**). * *p* < 0.05.

We first evaluated the association between CD44 mRNA expression in lung cancer tissues and patient survival by using online microarray datasets [29]. High expression of CD44 mRNA in lung cancer tissues was associated with poor overall survival of patients (Figure 1A). From immunohistochemical analysis, it was found that lymph nodes with metastasis showed higher CD44 protein expression compared to primary tumors (Figure 1B). We further analyzed CD44 expression in primary tumors and paired lymph nodes with metastasis by using flow cytometry and found that in the samples we tested, CD44 positive cells were enriched in the metastatic lymph nodes compared to the primary tumors (Figure 1C).

### 3.2. The Migration and Invasion Abilities of Lung Cancer Cells Were Reduced after CD44 Knockdown In Vitro

**Figure 2:**
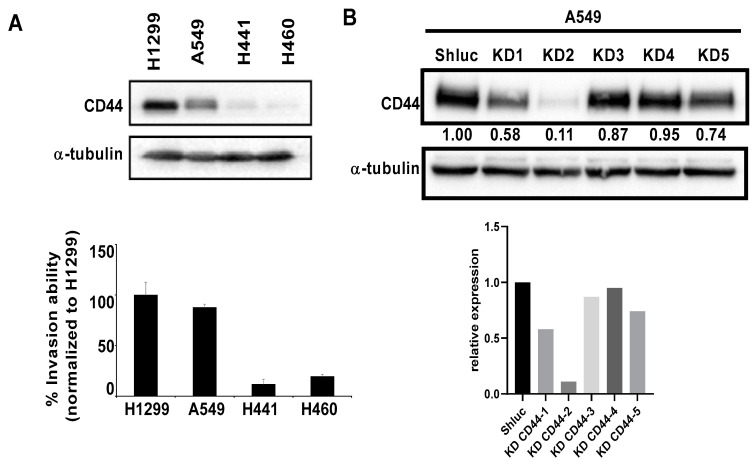

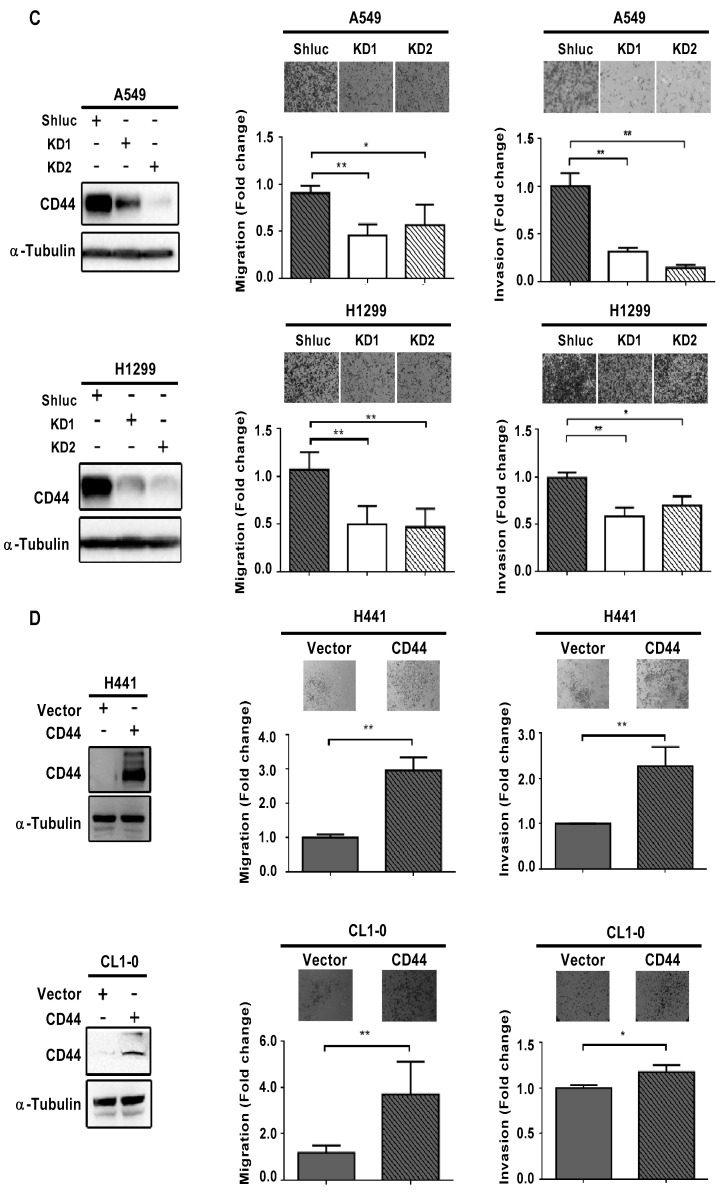
CD44 knockdown reduced migration and invasion abilities of lung cancer cells in vitro. (**A**) Expression of CD44 in different lung cancer cell lines and quantification of western data. (**B**) CD44 knockdown efficiency of different lentivirus clones and quantification of western data. (**C**) CD44 knockdown efficiency in A549 and H1299 cells determined by Western blot (**left**), and effects of CD44 knockdown on Transwell migration (**middle**) and invasion (**right**) abilities of A549 and H1299 cells. (**D**) CD44 overexpression efficiency in H441 and CL1-0 cells (**left**), and effects of CD44 overexpression on Transwell migration (**middle**) and invasion (**right**) abilities of H441 and CL1-0 cells. * *p* < 0.05, ** *p* < 0.01.

**Figure 3:**
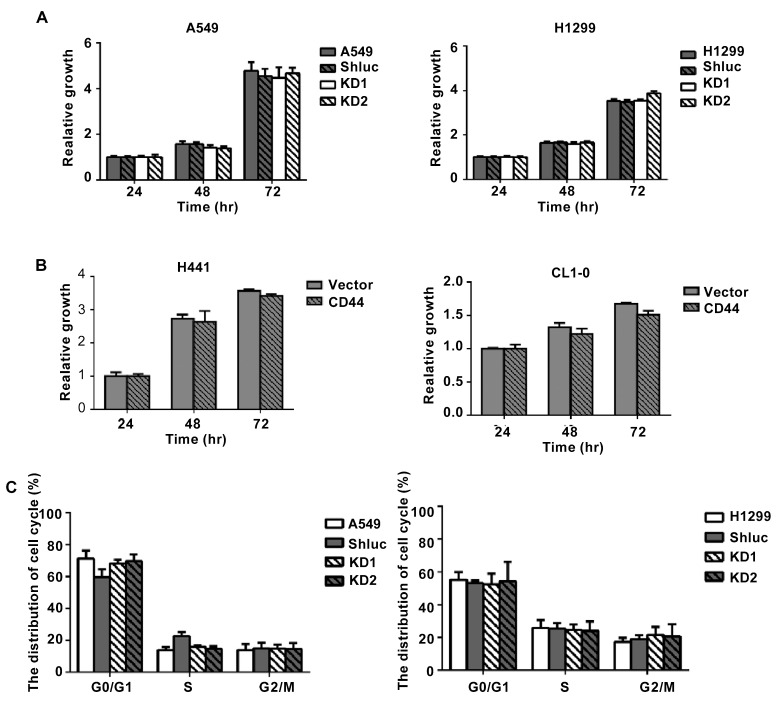
CD44 expression has no significant effects on cell proliferation and cell cycle. (**A**) Cell viability analysis in A549 and H1299 cells with CD44 knockdown. (**B**) Cell viability analysis in H441 and CL1-0 cells with CD44 overexpression. (**C**) Cell cycle analysis in A549 and H1299 cells with CD44 knockdown.

As IHC results revealed that CD44 might play a role in lung cancer metastasis, we further evaluated the expression of CD44 in different cancer cell lines and found that there was a positive correlation between CD44 expression and cancer cell invasion ability (Figure 2A). Next, we used overexpression and knockdown approaches to investigate the effects of CD44 on lung cancer cell migration and invasion abilities. We used five different clones for CD44 knockdown, and two of them with high knockdown efficiency were used for further experiments (Figure 2B). We overexpressed CD44 in lung cancer cell lines with low expression of CD44 and knocked down CD44 expression in lung cancer cell lines with high CD44 expression. The effects of CD44 overexpression and knockdown on cell migration and invasion abilities were investigated by using Transwell migration and invasion assays. We found that in CD44-knockdowned cell lines A549 and H1299, migration and invasion abilities were decreased (Figure 2C). On the other hand, in CD44-overexpressing cell lines H441 and CL1-0, migration and invasion abilities were increased (Figure 2D). We also investigated the effect of CD44 expression on lung cancer cell proliferation. The results showed that CD44 knockdown in A549 and H1299 cells and CD44 overexpression in H441 and CL1-0 cells had no effect on cell viability of lung cancer cells (Figure 3A,B). In addition, from PI staining results, the cell cycle distribution of CD44-knockdowned A549 and H1299 cells was similar to the control group (Figure 3C). These results suggest that CD44 promotes lung cancer cell migration and invasion abilities, but not cell proliferation.

### 3.3. CD44 Promotes Lung Cancer Cell Migration and Invasion through ERK Pathway

**Figure 4:**
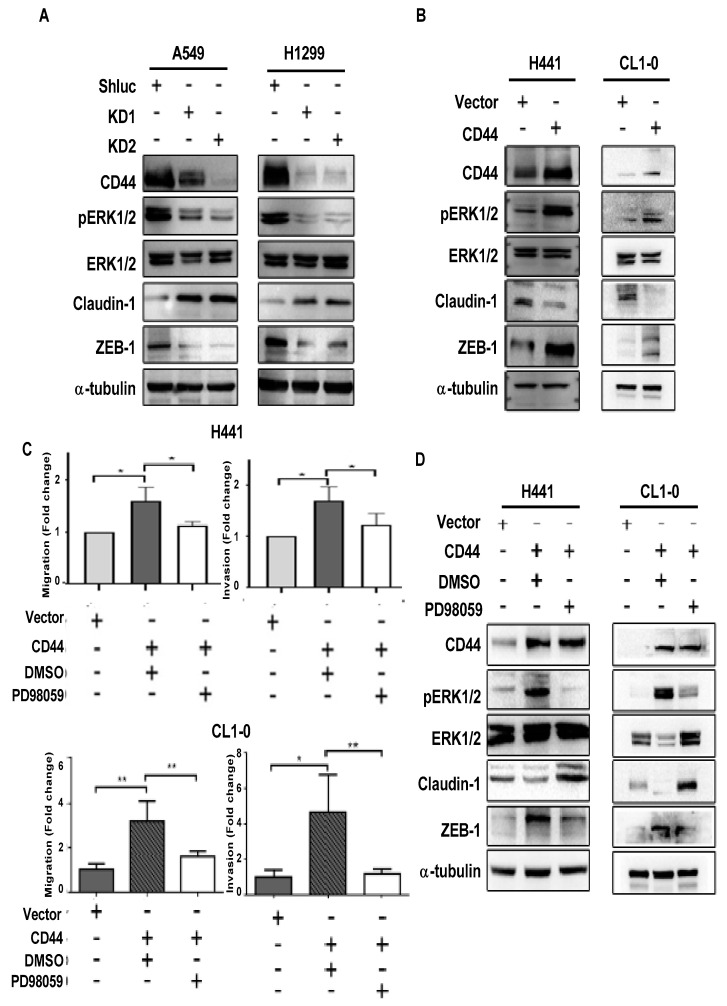
CD44 promoted migration and invasion abilities of lung cancer cells through ERK–ZEB1 signaling. (**A**) Western blot showing the expression pattern of p-ERK, ERK, Claudin-1, and ZEB1 in CD44-knockdowned A549 and H1299 cells. (**B**) Western blot showing the expression pattern of p-ERK, ERK, Claudin-1, and ZEB1 in CD44-overexpressing H441 and CL1-0 cells. (**C**) Effect of ERK inhibitor (PD98059, 25 uM) treatment for 48 h on migration and invasion abilities of CD44-overexpressing H441 and CL1-0 cells. (**D**) Western blot showing the expression pattern of p-ERK, ERK, Claudin-1, and ZEB1 in CD44-overexpressing H441 and CL1-0 cells after ERK inhibitor PD98059 (25 uM for 48 h) treatment. * *p* < 0.05, ** *p* < 0.01.

To further investigate the underlying mechanisms by which CD44 promotes lung cancer cell migration, we evaluated the protein expression of commonly involved pathways, including Akt, Jak/Stat, and ERK pathways, and found that ERK phosphorylation was upregulated in CD44-overexpressing cells. In CD44-knockdowned A549 and H199 cells, ERK1/2 phosphorylation was decreased (Figure 4A), whereas ERK1/2 phosphorylation was increased in CD44-overexpressing H441 and CL1-0 cells (Figure 4B). Since the ERK pathway has been reported to promote epithelial to mesenchymal transition (EMT) [30], we further examined the expression of EMT-related proteins in lung cancer cells with CD44 knockdown and overexpression. The results showed that epithelial marker Claudin-1 protein expression was significantly upregulated and mesenchymal marker ZEB1 protein expression was downregulated in CD44-knockdowned cells (Figure 4A), whereas Claudin-1 expression was decreased and ZEB1 expression was increased in CD44-overexpressing cells (Figure 4B). Moreover, migration and invasion abilities of lung cancer cells were reduced when they were treated with ERK inhibitor PD98059 (Figure 4C). In addition, ERK-inhibitor treatment in CD44 overexpressing cells also reversed Claudin-1 and ZEB1 expression (Figure 4D). These results suggest that CD44 promotes lung cancer cell migration through the ERK pathway.

### 3.4. Effect of CD44 on Lung Cancer Cell Metastasis In Vivo

**Figure 5:**
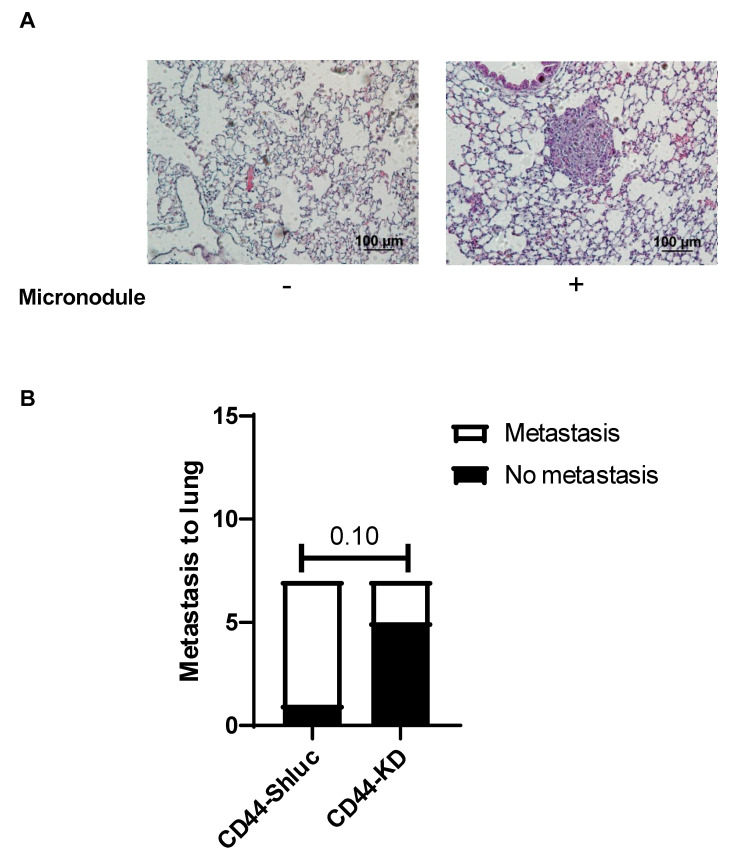
CD44 knockdown decreased lung cancer cell metastasis in tail-vein injection mouse model. (**A**) H & E staining showing representative photos for positive and negative metastatic nodules in the lung. (**B**) Bar graph showing the rate of lung metastasis in control and CD44 knockdown groups.

To investigate the effect of CD44 in vivo, we used the metastasis mouse model. We injected A549 CD44 control or knockdown cells into the tail vein of NOD SCID male mice and sacrificed the mice after 8 weeks to check for lung metastasis. Representative photos for negative and positive metastatic lung micronodules, determined by H & E staining, are shown in Figure 5A. We found that six out of seven mice showed metastasis to the lung in the control group, whereas only two out of seven mice showed lung metastasis in the knockdown group (Figure 5B). In addition, mice body weight was similar in both control (CD44-ShLuc) and CD44-KD groups during the study period of 8 weeks (Supplementary Figure S1). These results suggest that, indeed, CD44 promotes metastasis of lung cancer cells.

## 4. Discussion

CD44 has been reported to promote tumor progression, migration, and invasion by interacting with different proteins [13,31,32,33,34,35]. In this study, we conclude that CD44 promotes lung cancer cell metastasis in vitro and in vivo.

Using an online dataset, we found that CD44 overexpression is associated with poor survival of lung cancer patients. Moreover, IHC results from lung cancer patient tissue samples showed that CD44 expression was high in metastatic lymph nodes. In a previous meta-analysis study, it was reported that CD44 was associated with the migration ability of lung cancer cells [36]. CD44 positive cells from lung adenocarcinoma patients, compared to CD44 negative cells, have higher metastatic ability mediated by the Wnt/β–catenin–FoxM1–twist pathway [37]. Additionally, CD44 modulates the expression of transcription factor snail [38] and the mesenchymal phenotype in EGFR Kinase Inhibitors-resistant lung cancer [39]. In agreement with previous work, our in vitro and in vivo results show that CD44 plays a role in the metastasis of lung cancer cells. Furthermore, our study added a new insight regarding the role of CD44 in lung cancer metastasis that CD44 can promote lung cancer metastasis through the ERK–ZEB1 pathway.

ERK is a well-known protein that is involved in various biological processes, including cell proliferation, migration, and invasion of cancer cells [40,41]. In the current study, we found that ERK phosphorylation was increased in CD44-overexpressing cells and decreased in CD44-knockdowned cells. In agreement with our study, ERK and PI3K signaling have been reported to promote migration/invasion abilities of oral cancer cells through the expression of CD44 [42]. In addition, ERK inhibition has been reported to inhibit pancreatic cancer cell metastasis [43].

EMT marker analysis revealed that ZEB1 expression was increased, and Claudin-1 expression was decreased in CD44-overexpressing lung cancer cells. ZEB1 has been reported to promote the metastatic ability of different cancers, including lung cancer [44,45,46,47], while Claudin-1 is a metastasis suppressor in lung cancer [48]. In agreement with previous studies [30], we showed that ERK–ZEB1 signaling promotes epithelial to mesenchymal transition in cancer cells, and treatment of CD44-overexpressing cells with ERK phosphorylation inhibitor PD98059 reversed ZEB1 and Claudin-1 expression levels. These results indicate that CD44 promotes lung cancer migration and invasion through the ERK pathway by modulating ZEB-1 and Claudin-1 expression. Interestingly, a previous study also showed that ZEB1 is an upstream regulator of CD44 in human bronchial epithelial cells, which suggests that CD44 and ZEB may regulate each other’s expression in a cell type-dependent manner [49].

Our in vivo results revealed that lung metastasis rate was lower in mice injected with CD44-knockdowned lung cancer cells, although the *p*-value was only borderline significant from statistical analysis. Additionally, in our clinical study using primary lung tumors and paired lymph nodes, we were able to conclude that CD44 expression is positively correlated with lung cancer metastasis to regional lymph nodes. In summary, this study suggests that CD44 may be a potential prognostic marker and therapeutic target for lung cancer patients. Further extensive clinical studies using larger sample sizes are definitely required to consolidate our preliminary findings. An unsolved problem for this study is that CD44 has no effect on cell proliferation, which differs from a previous study showing that CD44 knockdown reduced cell proliferation in H460 lung cancer cells [50]. To solve the discrepant role of CD44 in lung cancer cell proliferation, BrDu incorporation assay can be included in future studies.

## 5. Conclusions

Based on our clinical, in vitro, and in vivo studies, this study provided evidence that CD44 promotes lung cancer metastasis through ERK/ZEB1 activation and may serve as a potential therapeutic target for lung cancer metastasis.

## Data Availability

The data presented in this study are available on request.

## Supplementary Materials

The following are available online at https://www.mdpi.com/article/10.3390/cancers13164057/s1, Figure S1: Body weight profile of the mice in control and CD44 knockdown groups.
