# Supplementary material for: CD44 Promotes Lung Cancer Cell Metastasis through ERK–ZEB1 Signaling
Source: Cancers (Basel). 2021 Aug 12;13(16):4057. doi: 10.3390/cancers13164057 (PMC8392539; doi:10.3390/cancers13164057)
Supplement: Supplementary file 1 [file cancers-13-04057-s001.zip › cancers-1313790 supplementary+wb/Raw data western_CD44.pptx]

## Slide 1
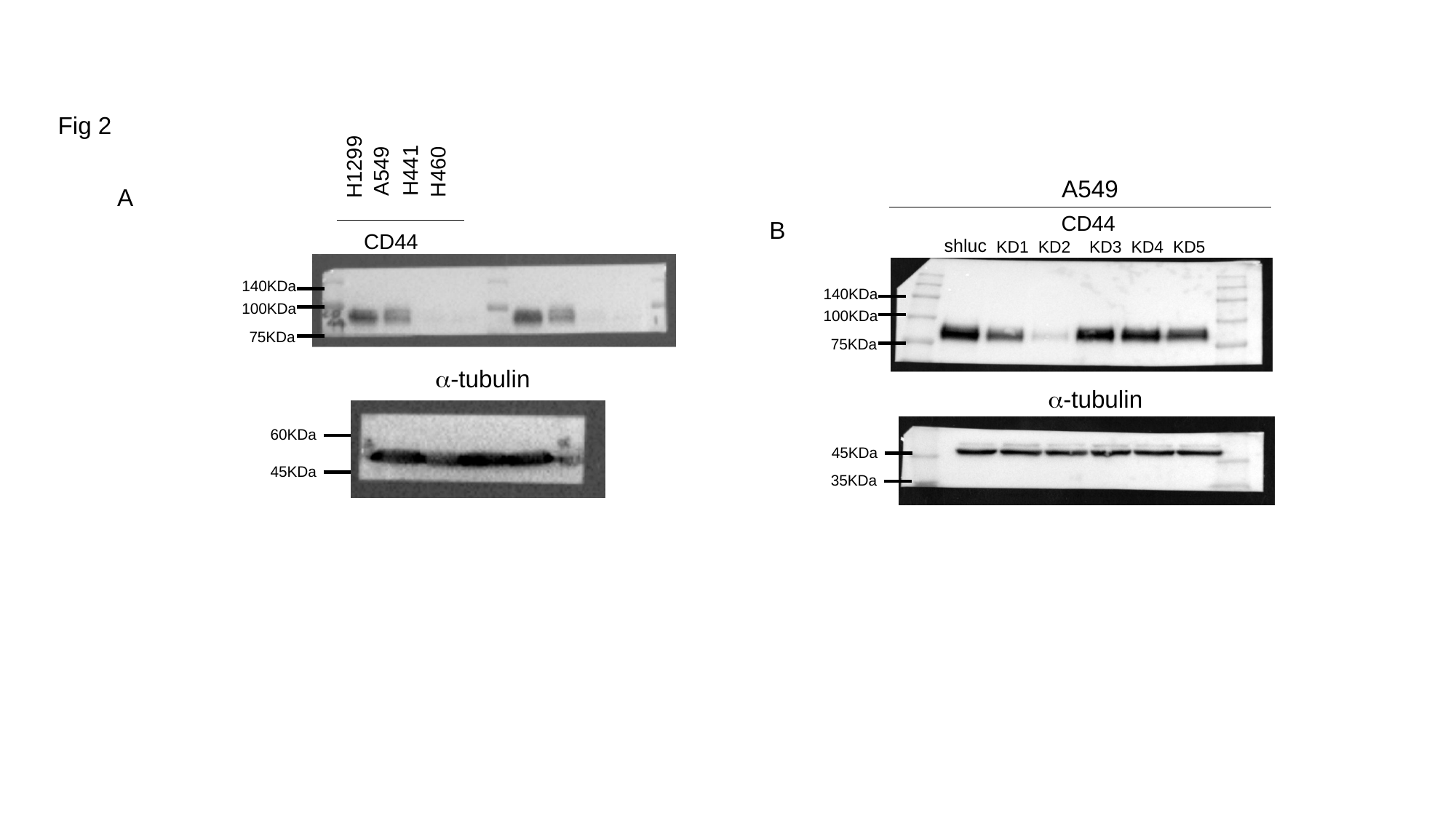

Fig 2
H1299
H441
A549
H460
A549
A
CD44
B
CD44
shluc
KD1 KD2 KD3 KD4 KD5
140KDa
140KDa
100KDa
100KDa
75KDa
75KDa
a-tubulin
a-tubulin
60KDa
45KDa
45KDa
35KDa

## Slide 2
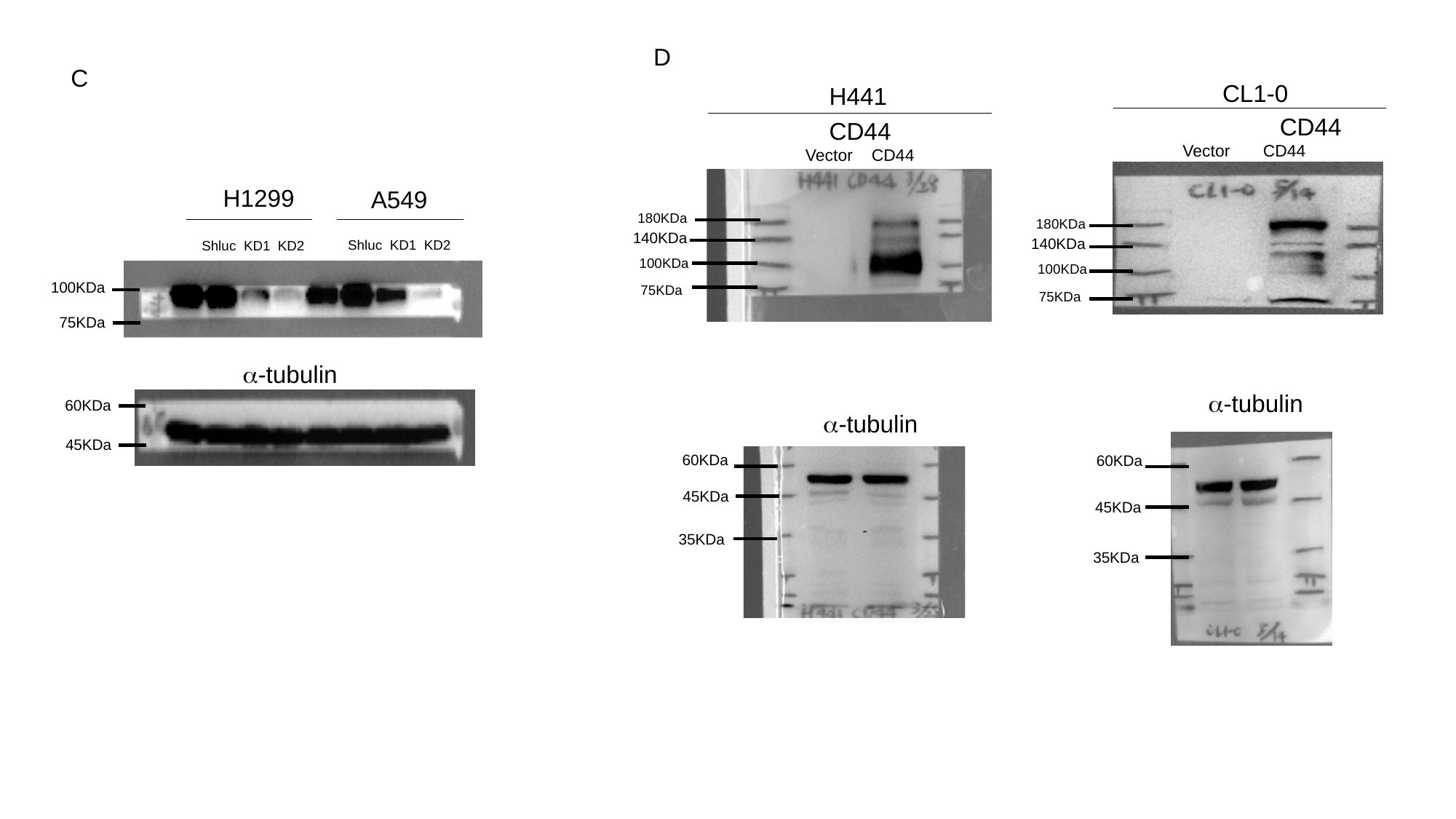

D
C
CL1-0
H441
CD44
CD44
Vector CD44
Vector CD44
H1299
A549
180KDa
180KDa
140KDa
140KDa
Shluc KD1 KD2
Shluc KD1 KD2
100KDa
100KDa
100KDa
75KDa
75KDa
75KDa
a-tubulin
a-tubulin
60KDa
a-tubulin
45KDa
60KDa
60KDa
45KDa
45KDa
35KDa
35KDa

## Slide 3
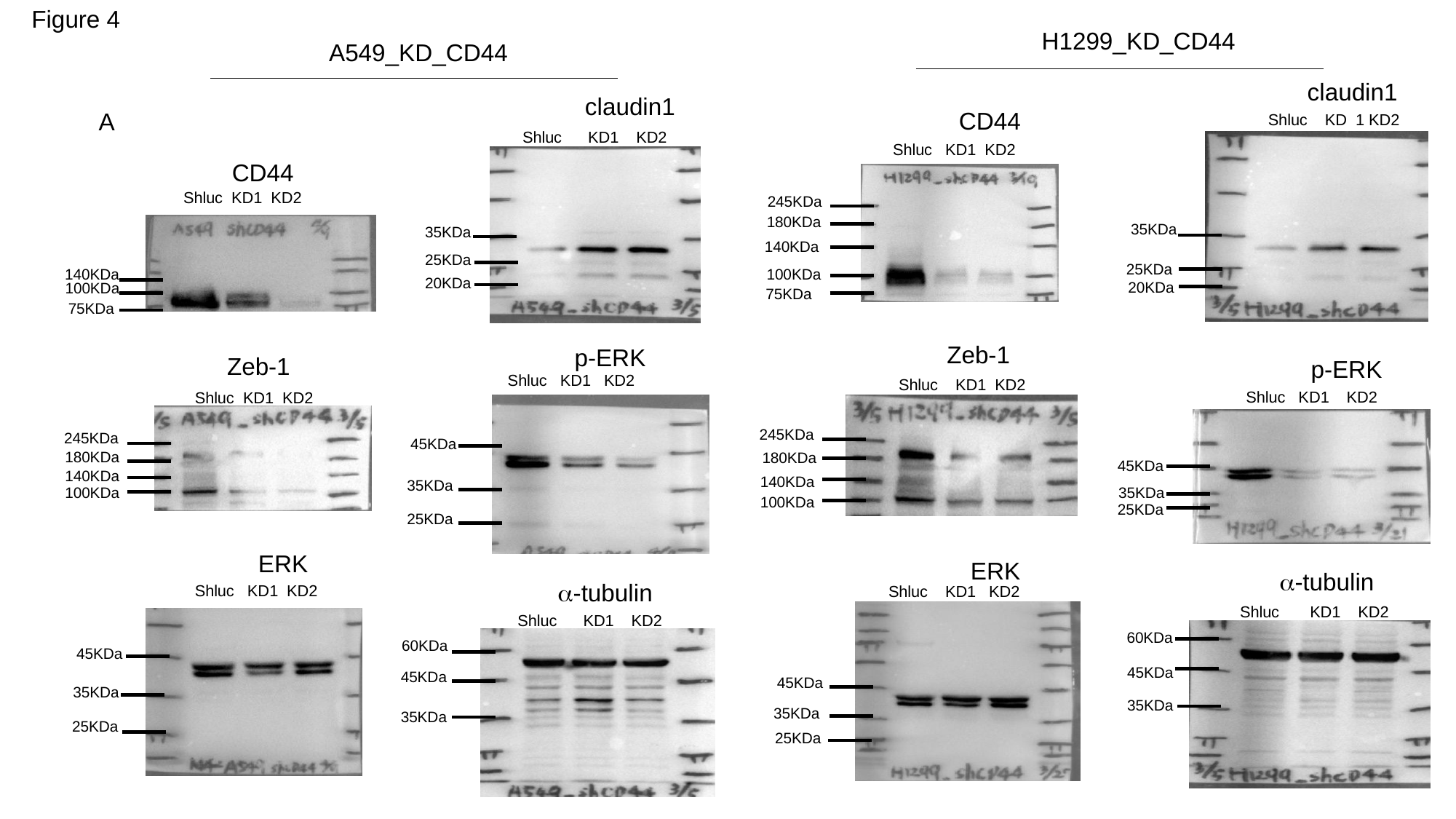

Figure 4
H1299_KD_CD44
A549_KD_CD44
claudin1
claudin1
CD44
A
 Shluc KD 1 KD2
 Shluc KD1 KD2
 Shluc KD1 KD2
CD44
 Shluc KD1 KD2
245KDa
180KDa
35KDa
35KDa
140KDa
25KDa
25KDa
140KDa
100KDa
20KDa
20KDa
100KDa
75KDa
75KDa
Zeb-1
p-ERK
Zeb-1
p-ERK
 Shluc KD1 KD2
 Shluc KD1 KD2
 Shluc KD1 KD2
 Shluc KD1 KD2
245KDa
245KDa
45KDa
180KDa
180KDa
45KDa
140KDa
140KDa
35KDa
35KDa
100KDa
100KDa
25KDa
25KDa
ERK
ERK
a-tubulin
a-tubulin
 Shluc KD1 KD2
 Shluc KD1 KD2
 Shluc KD1 KD2
 Shluc KD1 KD2
60KDa
60KDa
45KDa
45KDa
45KDa
45KDa
35KDa
35KDa
35KDa
35KDa
25KDa
25KDa

## Slide 4
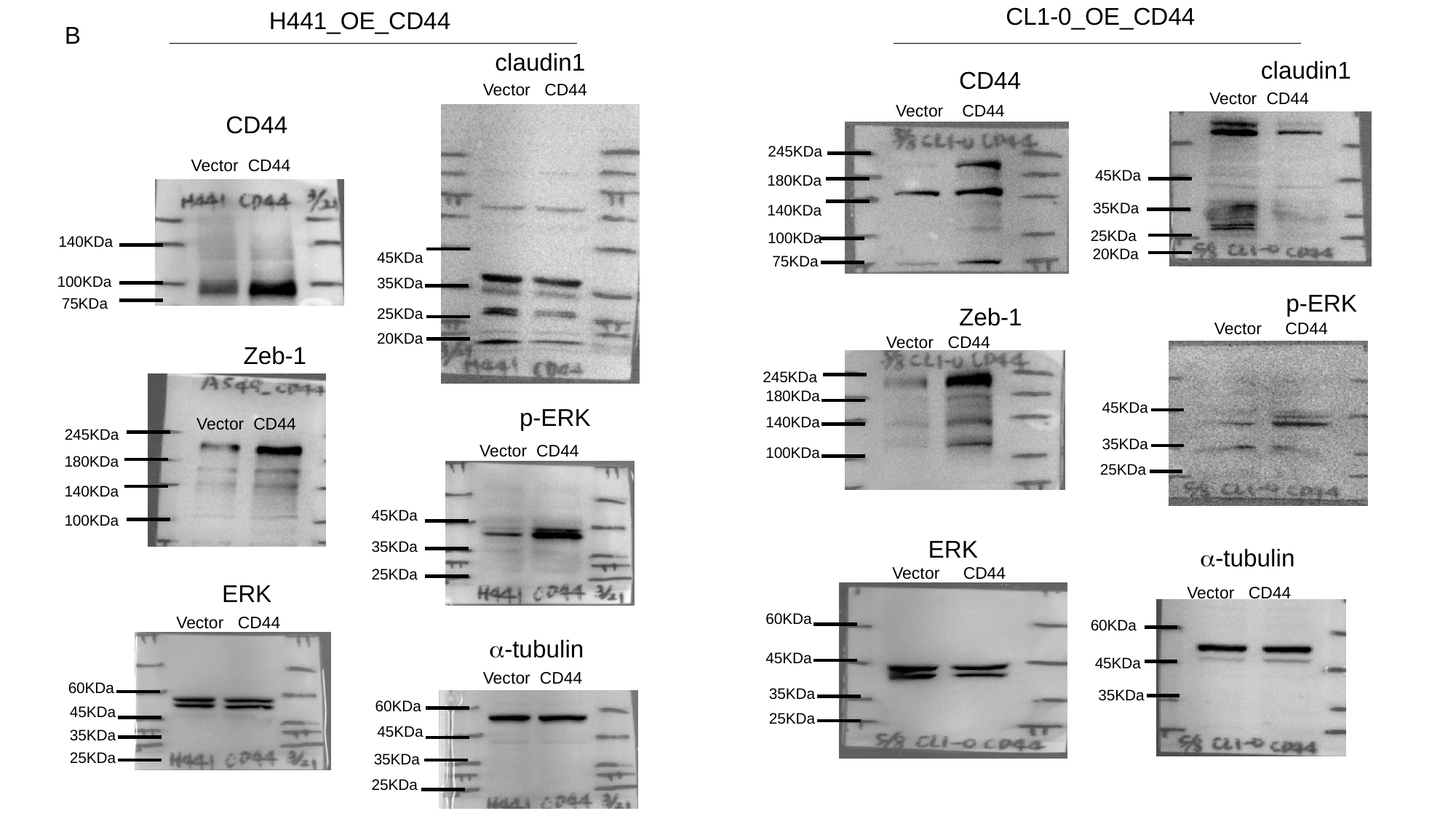

H441_OE_CD44
CL1-0_OE_CD44
B
claudin1
claudin1
CD44
Vector CD44
Vector CD44
Vector CD44
claudin1
CD44
245KDa
Vector CD44
45KDa
180KDa
35KDa
140KDa
25KDa
100KDa
140KDa
20KDa
45KDa
75KDa
100KDa
35KDa
p-ERK
75KDa
Zeb-1
25KDa
Vector CD44
20KDa
Vector CD44
Zeb-1
245KDa
180KDa
45KDa
p-ERK
140KDa
Vector CD44
245KDa
35KDa
Vector CD44
100KDa
180KDa
25KDa
140KDa
45KDa
100KDa
ERK
35KDa
a-tubulin
Vector CD44
25KDa
ERK
Vector CD44
60KDa
Vector CD44
60KDa
a-tubulin
45KDa
45KDa
Vector CD44
60KDa
35KDa
35KDa
60KDa
45KDa
25KDa
45KDa
35KDa
25KDa
35KDa
25KDa

## Slide 5
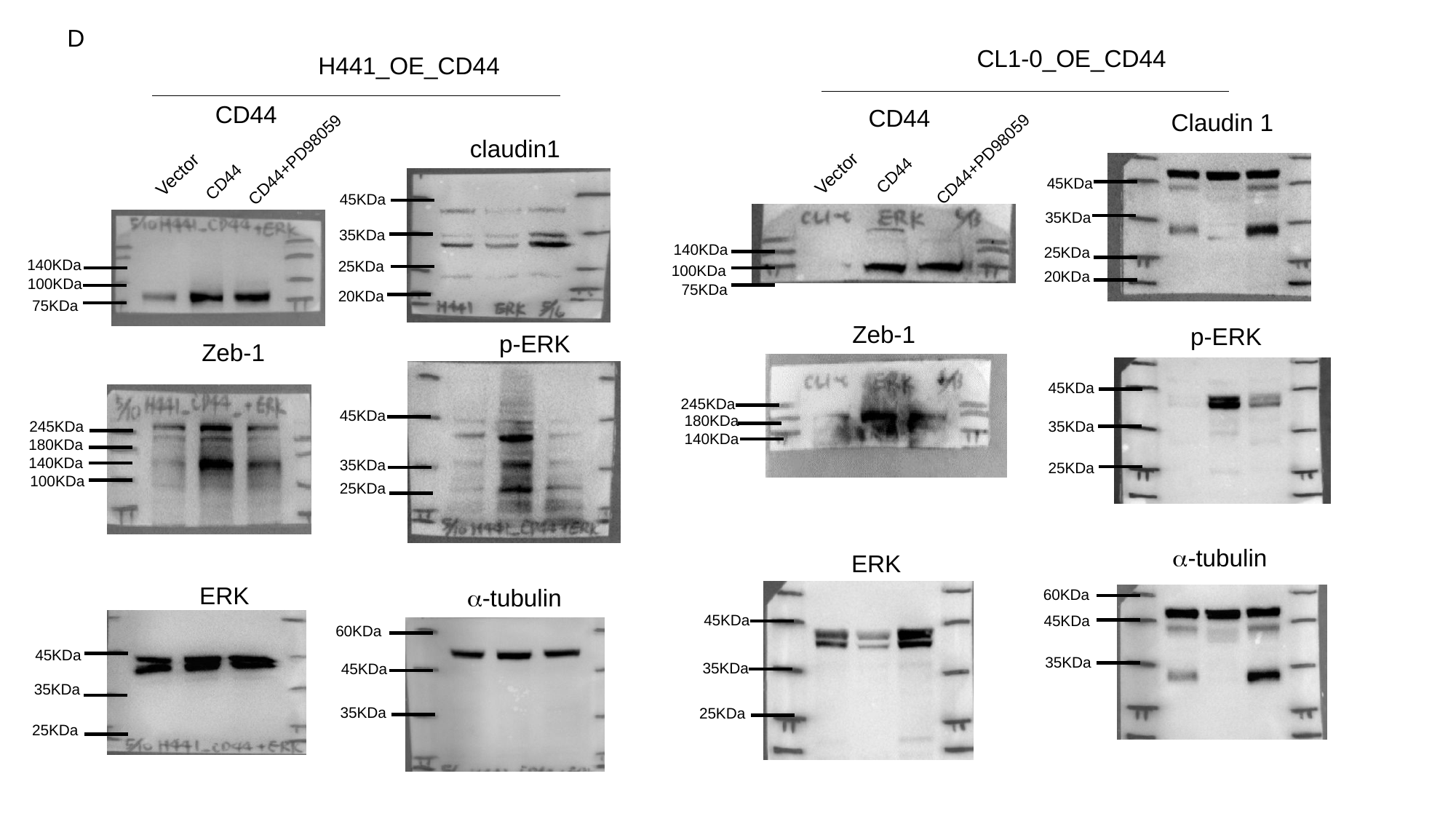

D
CL1-0_OE_CD44
H441_OE_CD44
CD44
CD44
Claudin 1
claudin1
CD44+PD98059
CD44+PD98059
Vector
Vector
CD44
CD44
45KDa
45KDa
35KDa
35KDa
140KDa
25KDa
140KDa
25KDa
100KDa
20KDa
100KDa
75KDa
20KDa
75KDa
Zeb-1
p-ERK
p-ERK
Zeb-1
45KDa
245KDa
45KDa
180KDa
35KDa
245KDa
140KDa
180KDa
140KDa
35KDa
25KDa
100KDa
25KDa
a-tubulin
ERK
ERK
a-tubulin
60KDa
45KDa
45KDa
60KDa
45KDa
35KDa
35KDa
45KDa
35KDa
35KDa
25KDa
25KDa
