# Supplementary figures and images for: CD44 Promotes Lung Cancer Cell Metastasis through ERK–ZEB1 Signaling
Source: Cancers (Basel). 2021 Aug 12;13(16):4057. doi: 10.3390/cancers13164057 (PMC8392539; doi:10.3390/cancers13164057)

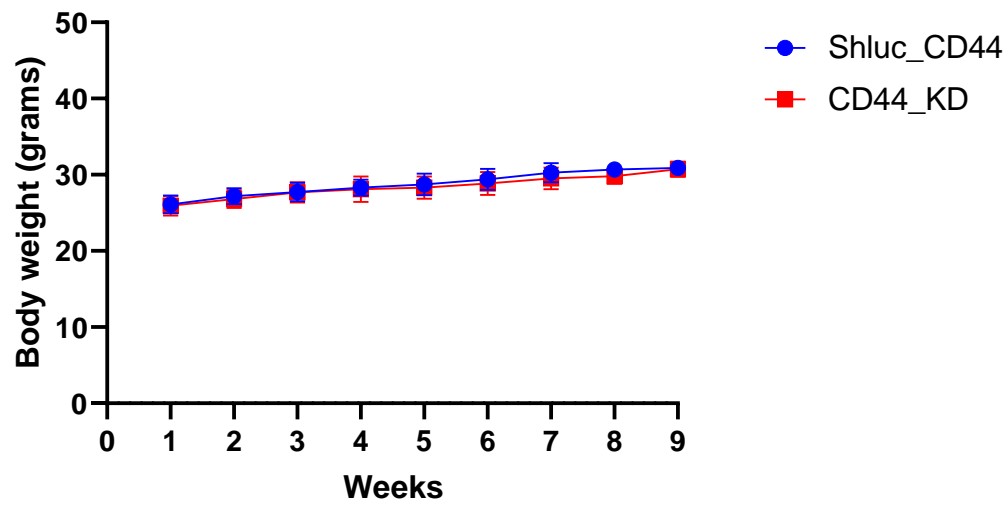

**Supplementary Figure 1. Body weight profile of the mice in control and CD44 knockdown groups.**

Supplement: Supplementary file 1 [file cancers-13-04057-s001.zip › cancers-1313790 supplementary+wb/cancers-1313790 supplemenatry .pdf]
